# What is a mental disorder? Evaluating the lay concept of Mental Ill Health in the United States

**DOI:** 10.1186/s12888-023-04680-5

**Published:** 2023-04-03

**Authors:** Jesse S. Y. Tse, Nick Haslam

**Affiliations:** Melbourne School of Psychological Sciences, University of Melbourne, Victoria, 3010 Australia

**Keywords:** DSM-5, Lay concepts, Concept breadth, Mental disorder, Mental illness, Mental health problem, Psychological issue

## Abstract

**Purpose:**

How “mental disorder” should be defined has been the focus of extensive theoretical and philosophical debate, but how the concept is understood by laypeople has received much less attention. The study aimed to examine the content (distinctive features and inclusiveness) of these concepts, their degree of correspondence to the DSM-5 definition, and whether alternative concept labels (“mental disorder”, “mental illness”, “mental health problem”, “psychological issue”) have similar or different meanings.

**Methods:**

We investigated concepts of mental disorder in a nationally representative sample of 600 U.S. residents. Subsets of participants made judgments about vignettes describing people with 37 DSM-5 disorders and 24 non-DSM phenomena including neurological conditions, character flaws, bad habits, and culture-specific syndromes.

**Results:**

Findings indicated that concepts of mental disorder were primarily based on judgments that a condition is associated with emotional distress and impairment, and that it is rare and aberrant. Disorder judgments were only weakly associated with the DSM-5: many DSM-5 conditions were not judged to be disorders and many non-DSM conditions were so judged. “Mental disorder”, “mental illness”, and “mental health problem” were effectively identical in meaning, but “psychological issue” was somewhat more inclusive, capturing a broader range of conditions.

**Conclusion:**

These findings clarify important issues surrounding how laypeople conceptualize mental disorder. Our findings point to some significant points of disagreement between professional and public understandings of disorder, while also establishing that laypeople’s concepts of mental disorder are systematic and structured.

## Background

One of the most vexed questions in the mental health field is how to define mental disorder [1]. This concept demarcates the conditions the field seeks to classify, understand, and treat. However, because these conditions are diverse and the boundary separating normality from pathology is fuzzy and unstable, it has been challenging to develop a definition that distinguishes which conditions should qualify as mental disorders and which should not. A clear definition would clarify the nature of mental disorder and adjudicate cases at the margins or at least help to clarify why some cases fall into a fuzzy boundary domain or are controversial.

A range of definitions of mental disorder has been put forward. Wakefield’s [2] influential harmful dysfunction account proposes that a mental disorder involves harm, in the form of distress and/or impairment, that is due to the failure of a psychological mechanism to perform its evolved function. Several writers [e.g., 1] have affirmed the centrality of harm to the concept, while others have challenged the dysfunction element of Wakefield’s definition [3]. Some critics have proposed that no strict definition is workable because mental disorder is a prototype-based concept [4]. More radical critics have argued that what we call mental disorder is in fact socially deviant behavior or ordinary problems in living rather than genuine medical illness [5]. *The Diagnostic and Statistical Manual of Mental Disorders (5th ed.; DSM-5)* [6] incorporates these concerns, defining a mental disorder as “a syndrome characterized by clinically significant disturbance in an individual’s cognition, emotion regulation, or behavior that reflects a dysfunction in the psychological, biological, or developmental processes underlying mental functioning”. This definition also specifies that social deviance or conflicts with society are not mental disorders unless they “result[] from a dysfunction in the individual” (p. 20) [6].

In addition to debating how mental disorder should be defined in the abstract, some authors have expressed concerns about how the concept is embodied in existing psychiatric classifications. These concerns focus on the *extensional* definition of mental disorder – the range of phenomena falling within it (e.g., the complete listing of recognized disorders), rather than on the necessary or sufficient conditions proposed by the *intensional* definition. A common critique in this work is that recent psychiatric classifications have become more expansive, either by including new disorders or by loosening the criteria for diagnosing existing disorders [7, 8]. This broadening process, variously referred to as medicalization, pathologization, disease-mongering, psychiatrization, concept creep, or diagnostic inflation [9–12], may have implications for overdiagnosis and overtreatment.

Given the theoretical and practical importance, and the longstanding debates about the definition of mental disorder, it is surprising that relatively little research has systematically addressed how the public understands the concept [13, 14]. There are extensive literatures in psychology, sociology, and anthropology on how specific mental health conditions are understood – often conceptualized in terms of idioms of distress [15], explanatory models of illness [16], lay theories [17], or folk psychiatry [18] – but very few studies have explored how the generic concept of mental disorder is defined (intensional definition) or the range of conditions that exemplify it (extensional definition).

How lay concepts of mental disorder align with or depart from professional concepts is a potentially fruitful line of inquiry, which could clarify important questions. For example, if laypeople understand mental disorder in terms of social deviance or abnormality rather than harm and dysfunction, this may help to make sense of the public stigma associated with it. Similarly, if laypeople’s concept of disorder is narrower than the concept embodied in psychiatric classifications, their reluctance to acknowledge the importance of some conditions or to seek help for them, might be clarified. Cultural and demographic variability in mental health-related behaviors might also be understood better if different cultures and demographic groups were shown to hold concepts of mental disorder that differ in content or inclusiveness.

Some prior research has addressed these questions. Haslam and Giosan [19] conducted a small study in which American undergraduates judged whether 68 vignettes – 47 describing *DSM-IV* [20] disorders and 21 describing conditions not recognized by *DSM-IV* – were mental disorders and rated a series of features that might predict those judgments. Participants tended to judge a narrower range of conditions to be disorders than the *DSM-IV* but *DSM-IV* conditions were much more likely to be seen as disorders than non-*DSM-IV* conditions, implying substantial alignment between lay and official concepts. Haslam and Giosan [19] also showed that participants judged conditions to be mental disorder primarily based on their perceived degree of harm (distress, impairment, and dysfunction) and abnormality (rarity and peculiarity). Further work found cross-cultural variations in disorder concepts [21, 22] and similar discrepancies in concepts of childhood disorders [23]. Together, these studies indicated that laypeople’s concepts of mental disorder may not align well with those advanced by theorists or embodied in psychiatric classifications such as the *DSM*. Lay concepts tended to be narrower than the *DSM* and varied across cultures. However, two decades had passed since these studies were conducted, during which *DSM* has been updated and lay concepts are likely to have evolved due to increased mental health awareness.

Some recent studies have continued to examine lay concepts of disorder. Rusch et al. [24] asked a large sample of English adults to judge whether six conditions, presented as labels rather than vignettes, were “a type of mental illness” (p. 643). Large majorities of participants strongly or slightly agreed that depression, schizophrenia, and bipolar disorder were mental illnesses, but much smaller proportions agreed for drug addiction, grief, and stress. Tikkinen and colleagues [25] conducted a similar study, asking a large Finnish sample, including laypeople, psychiatrists, physicians, and nurses, to judge whether 20 mental health-related states, identified by label only, were “diseases”. Laypeople had narrower concepts than the health professionals. For example, schizophrenia and depression were seen as diseases by all groups; grief and homosexuality were seen as diseases by none; and addictions and social anxiety disorder were seen as diseases by psychiatrists but not by laypeople. The Rusch et al. [24] and Tikkinen et al. [25] studies are valuable for addressing mental disorder-related judgments in large samples and for revealing differences between the judgments of laypeople and mental health professionals. However, both studies have limitations from the standpoint of characterizing lay concepts of disorder. First, they included only small samples of mental disorders, limiting the capacity to assess the extensional boundaries of the concept or its alignment with psychiatric classifications such as the *DSM*. Second, they did not explore the features participants used to make their disorder judgments, and therefore could not clarify the intensional content of the disorder concept. Third, by examining judgments of labels rather than vignette descriptions, the judgments may partially reflect familiarity with diagnostic terms as much as disorder concepts. Finally, Tikkinen et al.’s [25] use of “disease” terminology was generally considered outdated within psychiatry, and judgments of “disease” may not correspond to judgments of “mental disorder”.

This issue of terminology raises questions for previous research, in which different studies have asked participants to judge whether conditions were mental “disorders”, “illnesses”, or “diseases”. It is not yet known whether different terms such as these have different meanings for laypeople. In the present day, “mental disease” is rarely used, while “mental illness” is still current, although some writers object to its medical implications. “Mental disorder” was intended to be a more neutral substitute and less stigmatising than “mental illness” [26], but some people prefer expressions such as “mental health problem” that may have less severe connotations. These varied terms might have different levels of inclusiveness as well. “Mental illness” might refer to a narrower class of phenomena than “mental disorder” because its medical connotation might lead people to use it only in reference to conditions believed to have primarily biogenetic causes. “Mental health problem”, as a normalizing term, might be understood to refer to a broader and less severe range of phenomena than “mental illness” or “mental disorder”. Terms such as “psychological issue”, which lacks any direct implication of pathology or disturbance, may even be more inclusive. Determining whether laypeople ascribe similar or different meanings to alternative terms such as these is an important research question.

Building on this previous work, the present study aims to investigate multiple aspects of laypeople’s mental disorder-related concepts. Using a vignette-based methodology with a large U.S. nationally representative sample, it examines three fundamental research questions. First, it explores whether alternative generic terms (“mental disorder”, “mental illness”, “mental health problem”, and “psychological issue”) have similar or different meanings, both in which conditions are judged to best exemplify them and in the breadth of the respective concepts (i.e., the extension or range of conditions they include). Second, the study examines how well laypeople’s mental disorder-related concepts correspond to *DSM-5*, the currently dominant psychiatric classification. Third, it examines the intensional content of these concepts: the perceived features of psychological conditions that predict disorder judgments. The study is primarily descriptive and exploratory in nature, without explicit hypotheses. We anticipate that there would be differences in the meanings of the respective terms, that mental disorder-related concepts would show only a moderate alignment with the *DSM-5*, and that, as in previous studies [19], harm and abnormality judgments would predict which conditions are judged to be mental disorders.

## Method

### Participants

A U.S. nationally representative sample (stratified across age, sex, and ethnicity) of 623 participants was recruited from Prolific. Twenty-three participants (3.69%) were excluded due to incomplete responses, failing two or more of the three attention check questions [27], a completion time of less than 8 min [28], and/or straight-line responses. The final sample of 600 participants aged between 18 and 92 (*M* = 44.45, *SD* = 16.16) and contained 299 women, 291 men, eight non-binary people, and two who preferred not to say. It included 448 White Americans (74.7%), 81 Black or African Americans (13.5%), 45 Asian Americans (7.5%), and 26 others (4.3%).

### Materials

#### Vignettes

Sixty-one vignettes were written for this study, each referring to a person who might or might not be experiencing a mental disorder. Vignettes were two to five sentences long, and described a fictitious, unnamed person without identifying demographic information, unless this information was part of the diagnostic criteria of the condition in question. The vignettes did not label the conditions.

Of the 61 vignettes, 37 represented *DSM-5* conditions and 24 represented an assortment of non-*DSM-5* conditions. The 37 *DSM-5* conditions were sourced from all 19 broad classifications of the *DSM-5*, containing two from each classification except for one single-condition classification (gender dysphoria). The 24 non-*DSM-5* conditions were sampled using six heuristic categories drawn from the appendix of the *DSM-5* and previous studies [e.g., 21, 25]: character flaws, bad habits, medical/neurological conditions that may have a psychiatric aspect, conditions for further study listed in *DSM-5*, cultural syndromes, and other conditions. Four conditions were included for each category. The full list of conditions is presented in Table 1.

**Table 1:** List of All *DSM-5* and Non-*DSM-5* Conditions

Category	Condition
Non-*DSM-5*	
Character flaws	Recurrent cheating, jealousy, malingering, selfishness
Bad habits	Procrastination, poor hygiene, social media disorder, chronic lateness
Medical/neurological conditions	Migraine headache, chronic fatigue syndrome, multiple sclerosis, prosopagnosia
Conditions for further study in the *DSM-5*	Internet gaming disorder, caffeine use disorder, persistent complex bereavement disorder, suicidal behavior disorder
Cultural syndromes	Koro, mental disorder due to qigong, dhat, hikikomori
Other conditions	Obesity, midlife crisis, imposter syndrome, low self-esteem
*DSM-5*	
Neurodevelopmental disorders	Social communication disorder, intellectual developmental disorder
Schizophrenia spectrum and other psychotic disorders	Schizophrenia, schizoaffective disorder
Bipolar and related disorders	Bipolar I disorder (manic episode), cyclothymia
Depressive disorders	Major depressive disorder, persistent depressive disorder
Anxiety disorders	Social anxiety disorder, generalised anxiety disorder
Obsessive-compulsive and related disorders	Hoarding disorder, obsessive compulsive disorder
Trauma- and stressor-related disorders	Reactive attachment disorder, posttraumatic stress disorder
Dissociative disorders	Dissociative identity disorder, dissociative amnesia
Somatic symptom and related disorders	Factitious disorder, somatic symptom disorder
Feeding and eating disorders	Avoidant/restrictive food intake disorder, binge eating disorder
Elimination disorders	Enuresis, encopresis
Sleep-wake disorders	Insomnia disorder, restless legs syndrome
Sexual dysfunctions	Delayed ejaculation, female orgasmic disorder
Gender dysphoria	Gender dysphoria
Disruptive, impulse control, and conduct disorders	Conduct disorder, kleptomania
Substance use and addictive disorders	Gambling disorder, caffeine withdrawal disorder
Neurocognitive disorders	Delirium, mild neurocognitive disorder
Personality disorders	Narcissistic personality disorder, avoidant personality disorder
Paraphilic disorders	Sexual masochism disorder, exhibitionistic disorder

#### Label rating task

Participants were randomly allocated either to a label rating or feature rating task. Those who completed the label rating task read all 61 vignettes in randomized order and rated the extent to which each condition was an example of one of the four (randomly assigned) labels: “mental illness”, “mental disorder”, “mental health problem”, or “psychological issue”. The item “This person has a [label]” was rated on a 6-point Likert scale (1 = *strongly disagree* to 6 = *strongly agree*).

#### Feature rating task

Participants who completed the feature rating task rated a random subset of 13 of the 61 vignettes on 11 items representing features that might be associated with the concept of mental disorder. These features were drawn widely from theoretical analyses of the concept of mental disorder and previous research [19]. Features and corresponding items were as follows: Emotional distress (“This person is experiencing a lot of emotional distress”); impaired functioning (“This person has impaired functioning in everyday life”); severity (“The condition is severe”); need for treatment (“This person needs psychiatric treatment”); personal responsibility (“This person is responsible for the condition”); social aspect (“This condition only affects the person described, but not the people around them” [reversed]); stigma (“Most people would want to stay away from this person”); rarity (“This condition is rare”); normality (“This condition is experienced by everyone to some extent”); environmental cause (“The condition is caused by the person’s environment and life experiences”); biogenetic cause (“The condition is caused by genetic or other biological factors”). Participants rated their subset of conditions on the item “To what extent do you agree or disagree with the following statements describing the person above?” on a 7-point Likert scale (1 = *strongly disagree* to 7 = *strongly agree*).

### Procedure

The study was approved by the University of Melbourne Human Research Ethics Committee. An advertisement was listed on the Prolific platform. The eligibility of participants was dependent on creating a nationally representative sample of the United States reflecting the demographic distribution of age, gender, and ethnicity based on the U.S. Census Bureau’s census data. Eligible and interested Prolific users were redirected to the Qualtrics survey platform where they were shown the Plain Language Statement and completed the consent form. Participants were randomly allocated into five subgroups. Four subgroups completed different versions of the label rating task, rating all 61 vignettes on one of the four alternative labels. One subgroup completed the feature rating task, rating a subset of the 61 vignettes on all 11 features. The label rating and feature rating task subgroups were sampled disproportionately to approximately equalize the duration of the task for participants. After completing the main study task, the survey collected demographic and other information, including age, gender, race, education level, income, political orientation, first language, English proficiency, and the number of years living in the United States. Participants were then debriefed and paid for completion.

## Results

All analyses were conducted on aggregated ratings to capture the average judgments of all participants on each task. As none of the research questions addressed individual differences in judgments but related instead to shared judgments of whether conditions are or are not mental disorders and of the features of those disorders, data aggregation was appropriate. Data from the four label rating subgroups represented mean ratings across 62–68 participants of the 61 conditions on the four alternative labels. Data from the feature rating subgroup represented mean ratings across 68–76 participants of the 61 conditions on the 11 features. Therefore, the final data set for analysis contained mean ratings of the 61 conditions on 15 variables (four labels and 11 features).

### Alternative labels

To investigate whether the four alternative labels had similar or different meanings, we examined whether the label ratings were correlated across the 61 vignettes and whether they differed in mean rating (i.e., whether some labels were more inclusive than others). Table 2 presents the mean ratings of each label and the correlations between them. These correlations were extremely high, indicating that the same conditions were consistently rated as better or worse examples of all four labels. To evaluate differences in concept breadth, a one-way ANOVA (*n* = 61) was conducted to compare the mean ratings across the 61 conditions of the four labels. There was a significant difference between the labels, *F*(3,257) = 8.68, *p* < .001. Tukey’s HSD test indicated that “psychological issue” received higher mean ratings than “mental illness” (*p* < .001), “mental disorder” (*p* < .001), and “mental health problem” (*p* = .002), but these three labels did not significantly differ from one another. Thus, a higher proportion of the conditions were judged to exemplify “psychological issue” than the other three labels. Taking a mean rating of 3.5 (the midpoint on the disagree-agree scale) as a threshold, 32 of the 61 conditions were rated as “mental illnesses”, 35 as “mental disorders” and “mental health problems”, and 43 as “psychological issues”. “Psychological issue”, while having very similar conceptual content to the other labels, referred to a broader concept than the other labels. Overall, 32 conditions were judged to be examples of all four labels, 10 additional conditions were judged to be examples of at least one label, and 19 conditions were judged not to be examples of any label.

**Table 2:** Descriptive Statistics and Correlations for Label Ratings

Label	*n*	*M*	*SD*	Correlation			
				1	2	3	4
1. “Mental illness”	68	3.50	0.56	-	0.97***	0.97***	0.94***
2. “Mental disorder”	62	3.57	0.53		-	0.97***	0.95***
3. “Mental health problem”	64	3.59	0.53			-	0.97***
4. “Psychological issue”	67	3.93	0.51				-

***p < .001

### Correspondence with *DSM-5*

**Figure 1:**
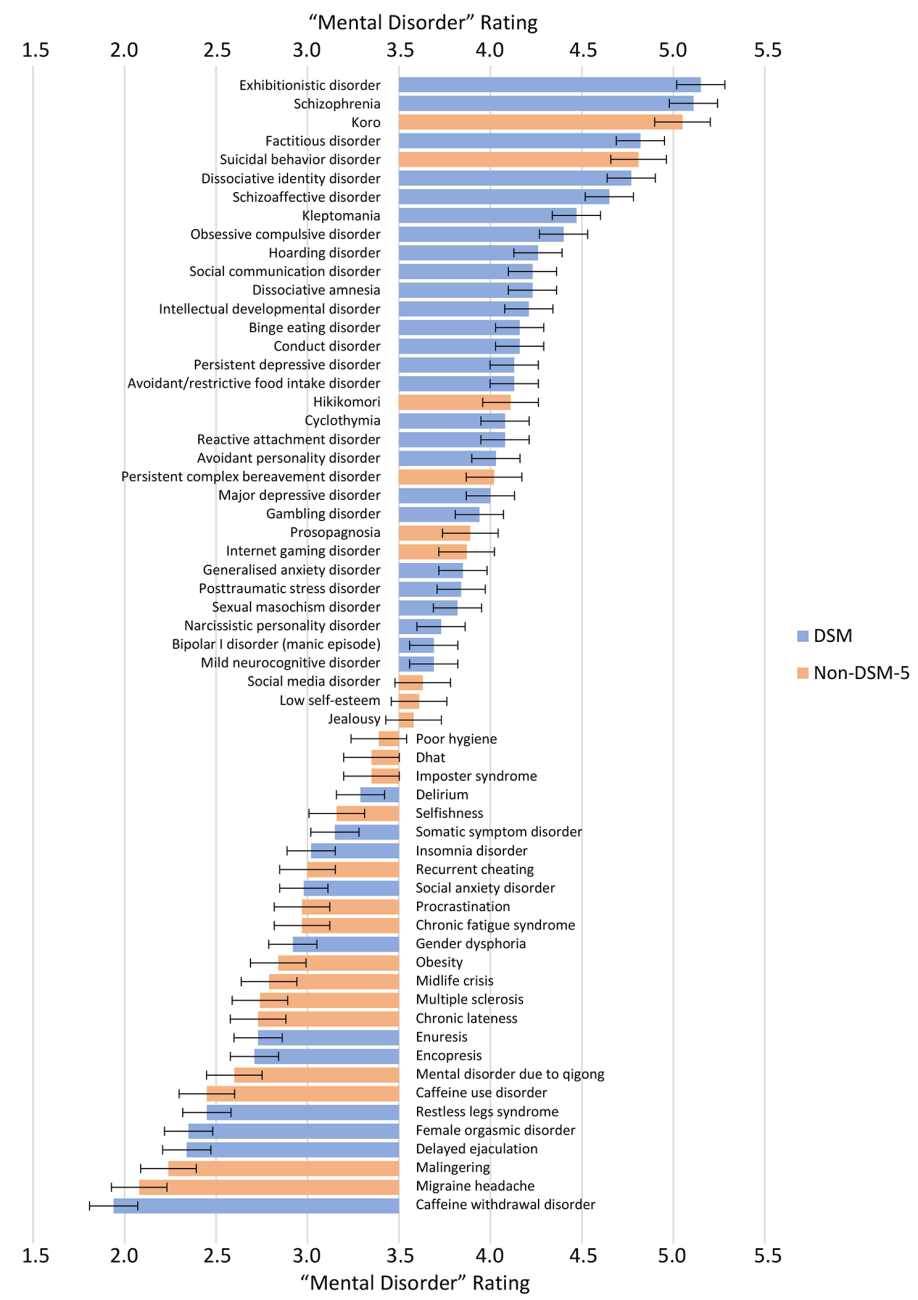
“Mental Disorder” Ratings for all 61 Conditions in Ranked Order

To determine the degree to which participants’ concepts corresponded extensionally to the *DSM-5*, we compared the mean ratings on the four labels between the 37 *DSM-5* conditions and the 24 non-*DSM-5* conditions. Vignettes representing *DSM-5* conditions were consistently rated higher than those representing non-*DSM-5* conditions, but this difference only reached significance for the “mental disorder” label, *t*(59) = 2.13, *p* = .038, implying weak correspondence. Eleven of the 37 *DSM-5* conditions (i.e., social anxiety disorder, somatic symptom disorder, enuresis, encopresis, insomnia disorder, restless leg syndrome, delayed ejaculation, female orgasmic disorder, gender dysphoria, caffeine withdrawal disorder, delirium) were rated below 3.5 on the “mental disorder” item, and 11 out of 24 non-*DSM-5* conditions (i.e., jealousy, social media disorder, prosopagnosia, intellectual developmental disorder, internet gaming disorder, persistent complex bereavement disorder, suicidal behavior disorder, social communication disorder, koro, hikikomori, low self-esteem) were rated above 3.5. “Mental disorder” ratings of all conditions are shown in Fig. 1.

### Features of mental disorder concepts

To analyse the features associated with mental disorder judgments (i.e., the intensional content of the mental disorder concept), a principal component analysis (PCA) was conducted on the mean ratings of the 11 features on the 61 conditions. Parallel Analysis suggested a three-component solution while MAP and scree tests suggested a four-component solution; the latter option aligned better with theoretical dimensions and accounted for 85.89% of the variance. Promax rotation method was employed to allow for correlations among the components. Component loadings are presented in Table 3.

**Table 3:** Component Loadings From the Principal Component Analysis

Item	Component loading			
	1	2	3	4
Emotional distress	**0.89**	− 0.40	− 0.11	0.16
Impaired functioning	**0.86**	0.25	− 0.17	− 0.28
Severity	**0.80**	0.26	0.19	− 0.03
Needs treatment	**0.66**	0.28	0.30	0.34
Personal responsibility	**− 0.54**	**0.56**	− 0.10	0.30
Social aspect	0.17	**0.92**	− 0.24	− 0.05
Stigma	0.03	**0.87**	0.18	− 0.02
Rarity	0.06	− 0.28	**0.97**	− 0.01
Normality	0.09	− 0.18	**− 0.88**	0.13
Biogenetic cause	0.42	0.06	− 0.07	**− 0.86**
Environmental cause	0.36	0.02	− 0.22	**0.84**
Variance explained (%)	33.41	25.75	18.28	8.45

The four-component solution was clearly interpretable. The first component distinguished conditions that were judged to be severe, emotionally distressing, functionally impairing, and requiring psychiatric treatment, which roughly corresponded to a judgment of harm. The second component represented variations in the extent to which conditions were stigmatized, seen as the fault of the affected person, and judged to affect other people. It could be described as a stigma and blame dimension. The third component identified conditions that were rare and beyond the continuum of normal experience, whereas the fourth component related to etiology, differentiating conditions seen as environmentally rather than biogenetically caused. The second component was moderately correlated with the fourth, *r* = .36, *p* = .004, indicating that conditions seen as more environmentally than biogenetically caused were typically more stigmatized.

Ratings of the 61 conditions on each of the labels were regressed on the four component scores of these conditions to evaluate whether people’s disorder judgments were based on the conditions’ perceived harmfulness, stigma, rarity, and etiology. The findings of the four analyses are summarized in Table 4. The four components powerfully predicted the ratings of each of the label ratings, explaining 73–75% of the variances. In addition, there were substantial similarities in the relative strength of the predictions of the four components. Component 1 (harm) was consistently the most strongly associated with disorder ratings for all four labels (semi-partial correlations with them ranged from 0.58 to 0.59) and component 3 (rarity) was the second strongest predictor for three of the labels. Components 2 and 4 added to the prediction of disorder judgments, albeit relatively weakly, except for component 4 being the second strongest predictor for “psychological issue”.

To clarify why “psychological issue” was more inclusive than the other labels, we compared component scores of the 10 conditions rated as “psychological issues” but not “mental illnesses” with those of the 31 conditions rated as both. The former group of conditions scored significantly lower on component 1, *t*(40) = -4.65, *p* < .001, and component 3, *t*(40) = -2.33, *p* = .025, suggesting that compared to other labels, “psychological issue” encompassed some conditions that were perceived as relatively low in harm and rarity.

**Table 4:** Summary of Multiple Regression Analyses for the Four Labels

Label	Model Summary			Unstandardised *B* of component			
	*R* ^2^	*F*(4,56)		1	2	3	4
Mental disorder	0.73	38.01***		0.48***	0.22***	0.26***	0.13*
Mental illness	0.75	42.03***		0.47***	0.19**	0.26***	0.14*
Mental health problem	0.75	42.85***		0.56***	0.24***	0.27***	0.22**
Psychological issue	0.75	41.09***		0.49***	0.18**	0.20***	0.27***

***p < .001, **p < .01, *p < .05

## Discussion

This study adds significantly to our understanding of the American public’s concepts of mental disorder. The findings speak to multiple aspects of these concepts, including the extent to which they vary according to different labels, their breadth (extension), their degree of resemblance to psychiatry’s concept of disorder, as institutionalised in the *DSM-5*, and the structure of features that underpin the disorder judgment (intension).

With regard to labels, our findings strongly suggest that different terms in widespread use are effectively synonymous, picking out nearly identical sets of conditions. Regarding breadth, one label (“psychological issue”) was more inclusive than the others, but overall, participants identified a substantial and very similar proportion of the conditions as mental disorders, mental illnesses, and mental health problems. Although the proportion of conditions participants identified as disorders was similar to the proportion identified as disorders by the *DSM-5*, there was only moderate overlap between the two sets of conditions. The public’s concept of disorder is not in lockstep with organized psychiatry. Finally, we found that our conditions were differentiated along dimensions of harm, stigma, rarity, and etiology, all of which were associated to varying degrees with disorder ratings. By implication, people judge whether a condition is a mental disorder primarily based on its degree of distress and impairment and its level of rarity and aberration.

These findings have implications for theory, research, and practice. How mental health conditions as a group should be labelled has been an ongoing source of debate, some may prefer “mental illness” or “mental disorder”, whereas others favour alternatives such as “mental health problem”. Our findings suggest that these concerns may be overblown because the three terms identified effectively identical sets of conditions and were grounded in the same feature judgments. “Mental illness” might have been expected to have a more medical reference than “mental disorder”, picking out a narrower set of more severe, biogenetically caused conditions, and “mental health problem” might have been expected to have a broader reference given the recent expansion of understanding of “mental health” itself, but no such differences emerged. Our findings indicate that laypeople do not meaningfully differentiate between several prominent labels and instead treat them as interchangeable. This conclusion is also compatible with the recent finding that alternative terms had little or no impact on stigma or the association between stigma and key outcomes [29].

The current findings imply that harm is central to the public’s concept of mental disorder as it is in influential theoretical analyses. Harm, in the form of distress and impairment, is present in *DSM-5*’s definition of mental disorder and Wakefield’s [30] analysis, and it also composed the set of features that most powerfully predicted the ratings of mental disorder in our study. However, our analysis suggests that laypeople’s disorder judgments reflect some elements that are not generally considered relevant to the definition of disorder by philosophically minded experts. Independent of harm severity, people were more likely to judge a condition to be a mental disorder if they perceived it as rare and unusual, stigmatized and blameworthy, and environmentally caused.

Most notably, judgments of rarity were potent predictors of disorder judgments, despite concerns that statistical abnormality should not be implicated in the mental disorder concept. Wakefield [2], for example, took issue with *DSM-III-R*’s [31] inclusion of statistical unexpectability as part of the definition of disorder. Our findings suggest that laypeople continue to consider statistical deviance or rarity as a feature of mental disorder, although their rarity judgments may represent an inference that an underlying dysfunction has occurred, per Wakefield’s analysis. There is evidence that judgments of internal dysfunction contribute to laypeople’s mental disorder judgments [32].

Our results may also have implications for practice. Attitudes to mental disorder and help-seeking are based on laypeople’s concepts of disorder, not directly on those held by professionals, and the disparities suggested by our findings may be significant. The only modest correspondence between public concepts of disorder and the *DSM-5* classification implies that laypeople believe that some “official” diagnoses are not legitimate disorders and that the official classification excludes some legitimate disorders. For example, with two exceptions (i.e., social anxiety disorder and gender dysphoria), the *DSM-5* disorders that our participants did not judge to be disorders involved specific somatically focused complaints (i.e., somatic symptom disorder, enuresis, encopresis, insomnia, restless leg syndrome, female orgasmic disorder, delayed ejaculation, and caffeine withdrawal disorder). By implication, laypeople tend not to view somatically focused complaints as falling within psychiatry’s purview. In contrast, our participants also tended to pathologize some conditions involving intense distress, behavioral addictions, or cultural syndromes that *DSM-5* does not recognize (e.g., persistent complex bereavement disorder, suicidal behavior disorder, internet gaming disorder, social media disorder, koro, hikikomori). Such discrepancies may contribute to misaligned help-seeking attitudes and behaviors, and consequently, conflicts between mental health professionals and the public over the former’s domain of expertise.

Nonetheless, the study has some limitations. Brief vignettes cannot fully capture the complexity of *DSM-5* criterion sets or the clinical significance criterion, so judgments that specific *DSM-5* conditions were or were not judged to be disorders must be interpreted with caution. The fact that these judgments stipulated “mental” or “psychological” – rather than being about “disorder,” “illness,”, “problem” or “issue” alone – might also have influenced them, potentially reducing ratings of conditions that lack an explicit mental aspect (e.g., “somatic symptom disorder”). Although it was highly predictive of disorder judgments, our set of features is likely to have missed some relevant elements in lay concepts of disorder. The features that distinguish *DSM-5* disorders from non-*DSM-5* conditions may also differ depending on the array of non-*DSM-5* conditions that is presented. Although our 24 non-*DSM-5* conditions were diverse and systematically sampled, a different pattern of disorder-linked features might be obtained if a different set of non-*DSM-5* conditions were used. Moreover, it should be noted that aggregated data of the kind employed in our analyses tend to yield stronger associations between variables than data based on individual judgments. “Ecological correlations” [33] based on mean ratings of this sort should not be interpreted as equivalent to the correlations that would be obtained between individuals’ ratings. The very strong associations obtained in our analysis are likely to overestimate the degree of predictability of mental disorder judgments at the level of individual participants. In addition, our use of aggregated data to study general patterns in public concepts of disorder is likely to have obscured individual, demographic, and cultural variabilities. Research has shown that individuals vary widely in the inclusiveness of their concepts of disorder [34]; and that different ethnic or racial groups also vary in the breadth of these concepts in ways that may be implicated in cultural differences in help-seeking [35]. Further research should examine systematic individual and cultural group differences in disorder concepts.

Despite these limitations, the present study goes some distance toward clarifying how laypeople conceptualize mental disorder, or at least the mix of concepts, theories, and indicators they employ when making mental disorder judgments. Although a very large literature has been amassed on public conceptions of specific conditions, the broad concept has been neglected, despite its relevance to enduring theoretical debates on the nature of mental disorder and practical issues regarding the public’s stigma and help-seeking. Our findings point to some significant points of disagreement between professional and public understandings of disorder, while also establishing that laypeople’s concepts of mental disorder are systematic and structured.

## Data Availability

The dataset analysed in the current study is available from the corresponding author on reasonable request.

## References

[1] Stein DJ, Palk AC, Kendler KS (2021). What is a mental disorder? An exemplar-focused approach. Psychol Med.

[2] Wakefield JC (1992). Disorder as harmful dysfunction: a conceptual critique of DSM-III-R’s definition of mental disorder. Psychol Rev.

[3] Bolton D. What is mental disorder?: an essay in philosophy, science, and values. Oxford University Press; 2008.

[4] Lilienfeld SO, Marino L (1999). Essentialism revisited: evolutionary theory and the concept of mental disorder. J Abnorm Psychol.

[5] Szasz TS (1960). The myth of mental illness. Am Psychol.

[6] American Psychiatric Association, Diagnostic and statistical manual of mental disorders. 5th ed. 2013, Washington, DC.

[7] Frances A (2013). Saving normal: an insider’s revolt against out-of-control psychiatric diagnosis, DSM-5, big pharma and the medicalization of ordinary life. Psychother Australia.

[8] Horwitz AV, Wakefield JC. The loss of sadness: how psychiatry transformed normal sorrow into depressive disorder. Oxford University Press; 2007. 10.1176/appi.ajp.2007.07081263 22688233

[9] Beeker T (2021). Psychiatrization of society: a conceptual framework and call for transdisciplinary research. Front Psychiatry.

[10] Brinkmann S. Diagnostic cultures: a cultural approach to the pathologization of modern life. Routledge; 2016.

[11] Conrad P, Slodden C. The medicalization of mental disorder, Handbook of the sociology of mental health. 2013,Springer.pp. 61–73.

[12] Haslam N (2016). Concept creep: psychology’s expanding concepts of harm and pathology. Psychol Inq.

[13] Bolton D (2010). Conceptualisation of mental disorder and its personal meanings. J Mental Health.

[14] Jaspers K. General psychopathology. Volume 2. JHU Press; 1997.

[15] Nichter M (1981). Idioms of distress: Alternatives in the expression of psychosocial distress: a case study from South India. Cult Med Psychiatry.

[16] Kleinman A. Patients and healers in the context of culture: an exploration of the borderland between anthropology, medicine, and psychiatry. Volume 3. University of California Press; 1980.

[17] Furnham A, Chan E (2004). Lay theories of schizophrenia. Soc Psychiatry Psychiatr Epidemiol.

[18] Haslam N (2005). Dimensions of folk psychiatry. Rev Gen Psychol.

[19] Haslam N, Giosan C (2002). The lay concept of “mental disorder” among american undergraduates. J Clin Psychol.

[20] American Psychiatric Association, Diagnostic statistical manual of mental disorders 4th ed. 1994, Washington, DC. 535.

[21] Giosan C, Glovsky V, Haslam N (2001). The lay concept of ‘mental disorder’: a cross-cultural study. Transcult Psychiatry.

[22] Glovsky V, Haslam N (2003). Acculturation and changing concepts of mental disorder: brazilians in the USA. Transcult Psychiatry.

[23] Giummarra MJ, Haslam N (2005). The lay concept of childhood mental disorder. Child Psychiatry Hum Dev.

[24] Rusch N, Evans-Lacko S, Thornicroft G (2012). What is a mental illness? Public views and their effects on attitudes and disclosure. Australian & New Zealand Journal of Psychiatry.

[25] Tikkinen KA (2019). Public, health professional and legislator perspectives on the concept of psychiatric disease: a population-based survey. BMJ Open.

[26] Fountoulakis KN. The concept and definition of mental illness, Psychiatry: From its historical and philosophical roots to the modern face. 2022,Springer.pp. 333–383.

[27] Goodman JK, Cryder CE, Cheema A (2013). Data collection in a flat world: the strengths and weaknesses of Mechanical Turk samples. J Behav Decis Mak.

[28] Zhang C, Conrad F (2014). Speeding in web surveys: the tendency to answer very fast and its association with straightlining. Surv Res Methods.

[29] Fox AB (2021). Mental illness, problem, disorder, distress: does terminology matter when measuring stigma?. Stigma and Health.

[30] Wakefield JC (1992). The concept of mental disorder: on the boundary between biological facts and social values. Am Psychol.

[31] American Psychiatric Association, Diagnostic statistical manual of mental disorders. 1987, Washington, DC.

[32] Wakefield JC (2006). The lay concept of conduct disorder: do nonprofessionals use syndromal symptoms or internal dysfunction to distinguish disorder from delinquency?. Can J Psychiatry.

[33] Robinson WS (2009). Ecological correlations and the behavior of individuals. Int J Epidemiol.

[34] McGrath MJ (2019). Concept creepers: individual differences in harm-related concepts and their correlates. Pers Indiv Differ.

[35] Tse JSY, Haslam N (2021). Inclusiveness of the concept of mental disorder and differences in help-seeking between Asian and White Americans. Front Psychol.

